# Flexible ZnO-mAb nanoplatforms for selective peripheral blood mononuclear cell immobilization

**DOI:** 10.1038/s41598-020-72133-0

**Published:** 2020-09-14

**Authors:** K. Sowri Babu, Pedro F. Pinheiro, Cátia F. Marques, Gonçalo C. Justino, Suzana M. Andrade, Marta M. Alves

**Affiliations:** 1 Division of Physics, Dept. Of Science and Humanities, Vignan’s Foundation for Science, Technology & Research (Deemed To Be University), Vadlamudi, Guntur, AP 522213 India; 2 Centro de Química Estrutural, Instituto Superior Técnico, Universidade de Lisboa, Av. Rovisco Pais, 1049-001 Lisboa, Portugal

**Keywords:** Sensors and biosensors, Immune cell isolation, Biophysical chemistry, NK cells

## Abstract

Cancer is the second cause of death worldwide. This devastating disease requires specific, fast, and affordable solutions to mitigate and reverse this trend. A step towards cancer-fighting lies in the isolation of natural killer (NK) cells, a set of innate immune cells, that can either be used as biomarkers of tumorigenesis or, after autologous transplantation, to fight aggressive metastatic cells. In order to specifically isolate NK cells (which express the surface NKp30 receptor) from peripheral blood mononuclear cells, a ZnO immunoaffinity-based platform was developed by electrodeposition of the metal oxide on a flexible indium tin oxide (ITO)-coated polyethylene terephthalate (PET) substrate. The resulting crystalline and well-aligned ZnO nanorods (NRs) proved their efficiency in immobilizing monoclonal anti-human NKp30 antibodies (mAb), obviating the need for additional procedures for mAb immobilization. The presence of NK cells on the peripheral blood mononuclear cell (PBMCs) fraction was evaluated by the response to their natural ligand (B7-H6) using an acridine orange (AO)-based assay. The successful selection of NK cells from PBMCs by our nanoplatform was assessed by the photoluminescent properties of AO. This easy and straightforward ZnO-mAb nanoplatform paves the way for the design of biosensors for clinic diagnosis, and, due to its inherent biocompatibility, for the initial selection of NK cells for autotransplantation immunotherapies.

## Introduction

Rapid and specific biological selection using inexpensive point-of-care systems is of the utmost importance in healthcare for early diagnosis, treatment, recovery and cure. Nanomaterials have stood out in the development of high performance and low-cost nanoplatforms, where zinc oxide (ZnO) stands out as a prominent nanomaterial due to its intrinsic physical and chemical properties. ZnO has a direct band gap of 3.37 eV, a large exciton binding energy of 60 meV, high electron mobility, and good chemical stability. Moreover, it also displays low toxicity and is a biologically compatible material^1, 2^. The isoelectric point (IEP) of ZnO, *ca.* 9.5, makes it suitable to adsorb negatively charged proteins, such as enzymes and antibodies, with lower IEPs^3^. As such, various types of biomolecules, including proteins^4^, enzymes^5^, antibodies^6^, as well as nucleic acids^7^, have been successfully bound to ZnO nanorods.

From the various types of ZnO nanostructures, such as nanoparticles, nanowires, nanoribbons, and nanoflowers, among others, nanorods (NRs) stand out for their higher surface area^8^. The chemical properties and organization of ZnO NRs can be modulated by the synthesis procedure, influencing their ability to adsorb biomolecules. When aiming for biosensor applications, ZnO NRs are typically prepared by a hydrothermal procedure^7, 9, 10^; however, this method requires the use of high temperature and pressure, limiting its use on flexible polymeric materials. Electrodeposition is an alternative and affordable method that can also deliver high-quality ZnO NRs^11^. This is a simple technique, which requires conducting substrates, uses eco-friendly aqueous electrolytes and allows a wide variation in the experimental conditions towards the optimization of ZnO NRs^12, 13^.

ZnO-based nanoplatforms are commonly used to adsorb low molecular molecules, like glucose^10^ and cholesterol^9^, but are also used to bind specific proteins, such as troponin^14^. ZnO nanoplatforms are also used, although to a smaller extent, to bind larger particles, such as viruses and bacteria^2, 7^. Interestingly, the use of immunoaffinity-based nanoplatforms, using monoclonal antibodies (mAb), to bind and select mammalian cells, while still relatively unexplored^15^, has a large potential for use in biomarker detection or as a tool for specific cell isolation. For instance, ZnO NRs platforms can be used for the selection of peripheral blood mononuclear cells (PBMC) subtypes, in particular of Natural Killer (NK) cells, which are part of the host innate immune cells. NK cells can be used in cancer diagnosis^15, 16^ and also have the ability to induce tumoral cell death, making them an interesting tool in immuno-oncology^15, 16^. As part of the immune system, NK cells can be used as a tool to fight cancer. For example, autologous cell transplantation has been used in clinical settings, including boosting the immune system function for immunotherapy approaches. In particular, the auto-transplantation of ex vivo-cultivated NK cells has led to an improved outcome in various cancer situations^17–20^.

One of the major issues in using NK cells for autologous transplantation is their isolation. NK cells make up 5 to 10% of all lymphocytes in circulation (*ca.* 1.5 to 3.0 × 10^3^ cells), and typically amount to 90 to 590 cells/mL of blood, thus being difficult to isolate^21, 22^. Recently, Tamashevski et al. used ZnO NR-immobilized fluorescein-labelled antibodies to detect lymphocytic leukaemia cells^15^. With the increase in the worldwide incidence of cancer, affordable and versatile tools for cancer detection and management are required. As such, to improve Tamashevski et al. system, non-labelled antibodies, affordable dyes and improved ZnO NR films can be used to convey a more effective and cheaper nanoplatforms on flexible substrates for NK cell isolation.

Acridine orange (AO), a common fluorescent dye, can be used to assess the status of PBMCs adsorbed in a nanoplatform. This dye, thoroughly used as a metachromatic stain for nucleic acids, has a fluorescence signal that strongly depends on pH. While AO intercalates DNA^23^, exhibiting fluorescence in the green region, it electrostatically binds RNA, displaying a fluorescence signal in the red region, above 630 nm, due to self-aggregation^24^. This red fluorescence, which is also observed when AO accumulates in the acidic intracellular compartments, such as lysosomes, has been used to distinguish apoptotic from necrotic cells^25^.

AO can be further used for cell imaging through confocal fluorescence lifetime imaging microscopy (FLIM) both in vitro and in vivo^26^. FLIM relies on the fluorophore lifetime sensitivity to the local environment or chemical state. Also, fluorescence lifetime is an intrinsic property of the fluorophore and does not depend on the fluorescence intensity, which is particularly important in scattering media.

In order to obtain an affordable and specific nanoplatform to select NK cells, we developed a flexible ZnO immunoaffinity-based nanoplatform. A well-ordered ZnO NR film was prepared by electrodeposition onto a flexible ITO (indium tin oxide)-coated PET (polyethylene terephthalate) (ITO/PET) substrate. To ensure the most selective approach for NK cell isolation from PBMCs, a monoclonal anti-human NKp30 antibody (mAb) was immobilized on the ZnO NR film. The selection of NK cells by this nanoplatform was assessed using AO-loaded PBMCs. NK cell identity was confirmed by stimulation with B7-H6, a specific NKp30 agonist that triggers cell degranulation, leading to extrusion of the AO dye; NK cell status was assessed by FLIM and SEM imaging.

## Results and discussion

### Physicochemical characterization of ZnO nanorods

**Figure 1:**
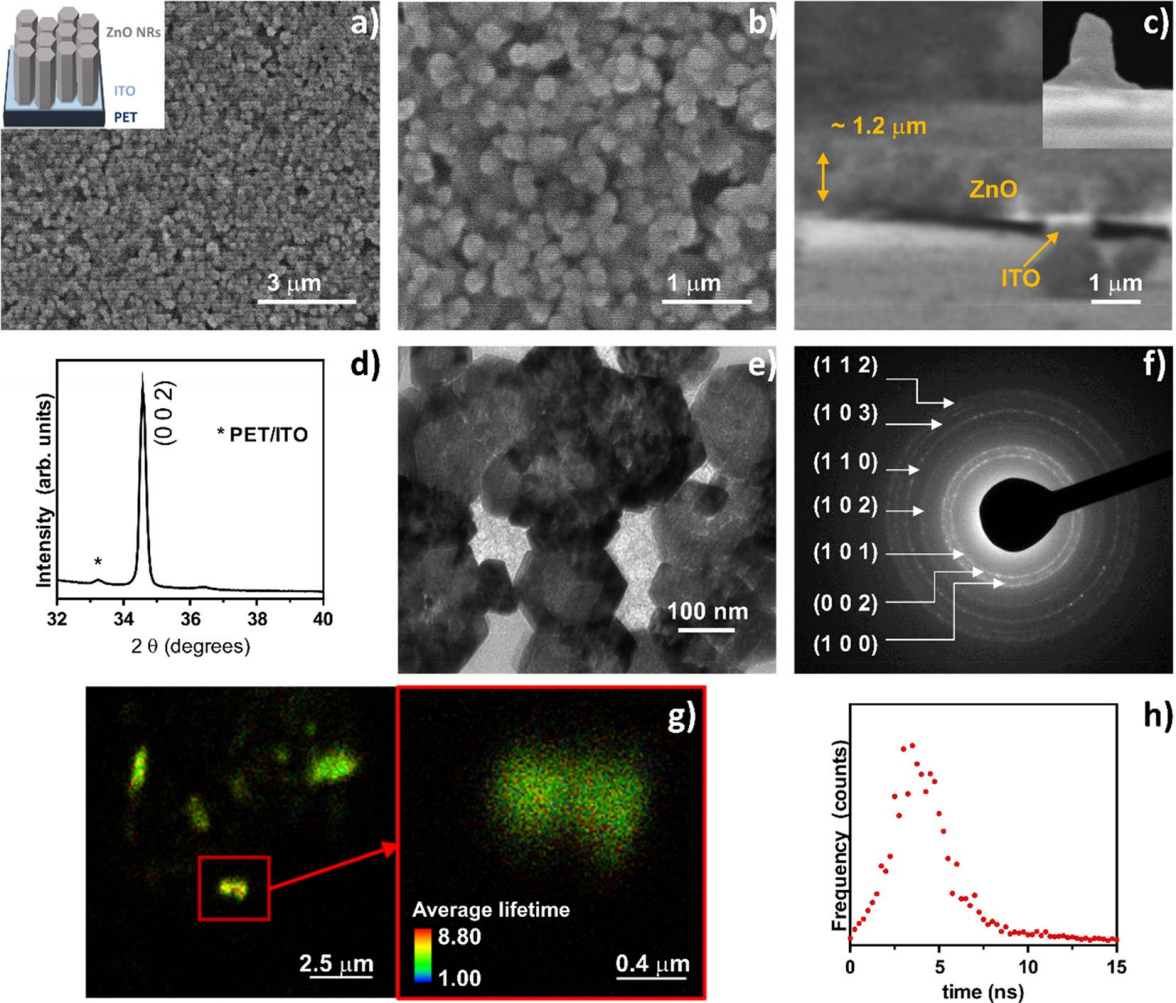
Physicochemical characterization of ZnO NRs. Scanning electron microscopy (SEM)—(**a**,**b**), top-view; (**c**) cross-section of the ZnO NRs film; a single ZnO nanorod on the ITO/PET substrate is shown in the inset; (**d**) X-ray diffractogram of the ZnO NRs film; (**e**) transmission electron microscopy (TEM) image of the ZnO crystals; (**f**) electron diffraction (ED) of crystal agglomerates; (**g**) fluorescence lifetime imaging microscopy (FLIM) image of ZnO NRs together with a zoomed-in image of the square region and the corresponding lifetime histogram (**h**).

The ZnO NR film obtained by electrodeposition was fully characterized, and the results are shown in Fig. 1. The structure of ZnO NRs was confirmed by scanning electron microscopy (SEM), where well-organized ZnO NRs, with a hexagonal top surface, are homogeneously distributed on the flexible Indium Tin Oxide/PolyEthylene Terephthalate (ITO/PET) substrate (Fig. 1a–c). This morphology agrees with the reported ZnO structures formed upon cathodic electrodeposition on transparent conducting substrates^11, 27^. Figure 1b shows that these hexagonal rods shape structures have varying diameters, below 600 nm. Similar diameters were reported by Lin et al.^28^ upon electrodeposition of ZnO NRs on ITO. The cross-section image of the ZnO film suggests that the film is composed by well-organized structures and has a total thickness *ca.* 1.2 µm (Fig. 1c). This result is in line with the height observed for an isolated ZnO rod (Fig. 1c, inset), suggesting that the film is formed by vertically aligned ZnO NRs, a typical arrangement achieved when using electrodeposition. The crystallinity of the film, assessed by the presence of the (0 0 2) reflection peak (Fig. 1d), illustrates that the ZnO nanorods grown on the PET/ITO substrate show a highly preferential orientation along the c-axis, perpendicularly to the substrate surface, in agreement with literature data^11, 29, 30^. A detailed view of ZnO crystals was achieved by TEM (Fig. 1e), where the typical hexagonal shape of ZnO crystals is visible. This image further illustrates the variable diameters of the nanorods, where hexagons with diameters as big as 300 nm and as small as 50 nm can be depicted. When analysing the electron diffraction (ED) pattern of ZnO NRs agglomerates, the presence of the ZnO crystalline planes (1 0 0), (0 0 2), (1 1 0), (1 0 2), (1 0 3) and (1 1 2) (Fig. 1f) confirms the presence of pure wurtzite crystals, as typically reported for these ZnO crystals^31^.

Profiting from the intrinsic photoluminescence (PL) of the ZnO NRs, fluorescence lifetime imaging microscopy (FLIM) was used to further characterize these structures, employing a laser excitation wavelength of 405 nm. The presence of several rod-like objects, with lengths of approximately 1.5 µm, was corroborated (Fig. 1g), and, upon zooming, some structures with sizes around 400 nm were observed (Fig. 1g). These results are compatible with the SEM measurements performed on the top view of vertically aligned NRs (Fig. 1b). The histogram associated with these FLIM images (Fig. 1h) indicates an average fluorescence lifetime of 5 ns, with low background contamination. This lifetime is in the same order of magnitude of previously determined values for ZnO^31^.

### PBMCs stimulation with B7-H6

**Figure 2:**
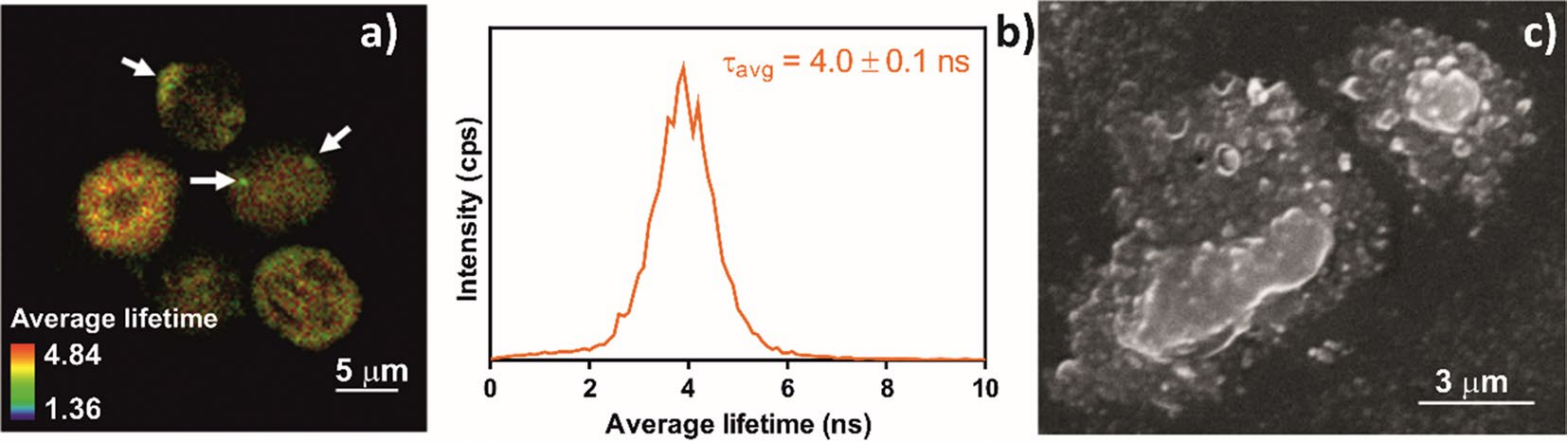
Characterization of PBMCs stimulation with B7-H6. (**a**) Fluorescence lifetime imaging microscopy (FLIM) image and (**b**) corresponding lifetime histogram; (**c**) scanning electron microscopy (SEM) image.

PBMCs, isolated from blood samples by Ficoll centrifugation, are a heterogeneous cell population that contains NK cells. NK cell presence in the isolated PBMC samples was confirmed by stimulating the isolated cells with B7-H6, a specific agonist of the natural cytotoxicity receptor NKp30 that is exclusively expressed in the membrane of NK cells. B7-H6 activation of the NKp30 receptor triggers NK cell degranulation, causing them to release the content of acidic cytotoxic granules. When cells are pre-loaded with acridine orange (AO), degranulation can be followed by tracking the fluorescent dye as well as by the changes in cell morphology (Fig. 2).

A clear FLIM image of activated NK cells was obtained, showing round-shaped objects of 5.5 × 6.5 µm^2^ (Fig. 2a). Typical NK cell degranulation was confirmed by the accumulation of AO in the outermost part of the cells instead of in the nuclei. The lifetime histogram, where an average lifetime of 4.0 ns can be observed (Fig. 2b), also indicates that AO aggregation is occurring, as such long lifetimes are linked to the prevalence of AO dimers or higher aggregates, which are concurrent with AO accumulation inside the granules. A deeper morphological analysis of the activated cells, made by SEM, revealed the altered morphology of these immune system cells^32^, which is in line with the formation of granules and their excretion from the cells (Fig. 2c). The successful degranulation of these cells upon B7-H6 stimulation confirms the presence of NK cells in the isolated PBMCs.

### Characterization of ZnO NRs, mAb and PBMC interactions

**Figure 3:**
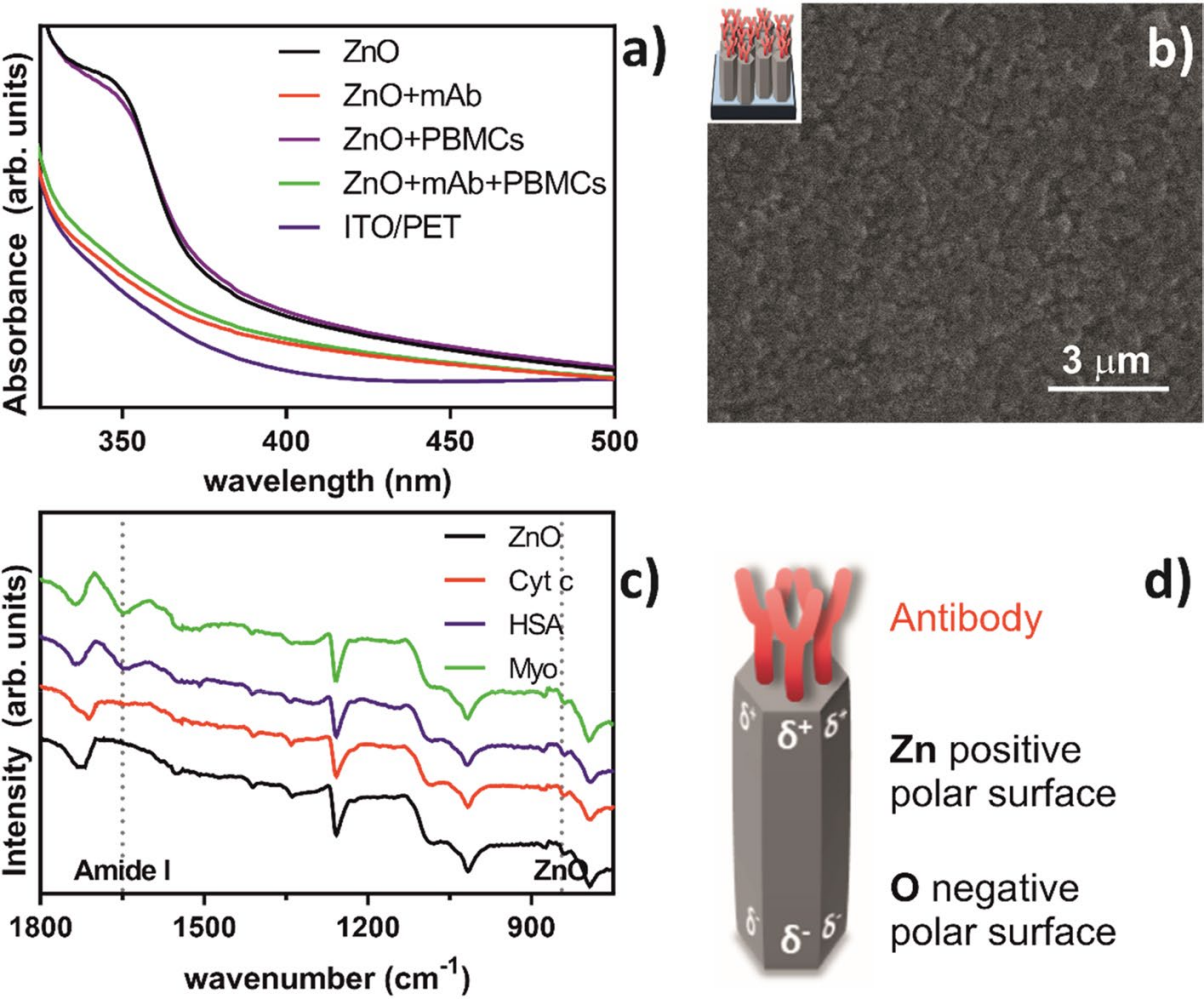
Characterization of the affinity of ZnO NRs film towards mouse anti-human anti-NKp30 monoclonal antibody (mAb); (**a**) UV–Vis spectra of the ZnO NRs film alone, together with the mAb, with or without PBMCs; (**b**) scanning electron microscopy (SEM) image of the immobilized mAb on the ZnO NRs film; (**c**) Fourier transform infrared attenuated total reflection (FTIR-ATR) of ZnO NRs film before and after incubation with three different proteins: myoglobin (Myo), human serum albumin (HSA) or cytochrome C (Cyt C); and (**d**) representative interaction of the immobilized mAb on ZnO NRs.

UV–vis absorption spectroscopy was employed to assess antibody immobilization on the surface of ZnO NRs. Figure 3a shows the UV–vis absorption spectra of ZnO NRs before and after the addition of antibodies and cells. Pristine ZnO NRs display a high UV absorption, with an absorption maximum at 360 nm that disappears upon incubation with the protein. This high UV absorption band, corresponding to the direct electron transition between the valence and the conduction bands, that steeply disappears below 400 nm, is characteristic of ZnO nanorods^33, 34^**.**

This peak remains absent upon incubation with cells, suggesting that the antibodies were preserved on ZnO surface even after the incubation with PBMCs. No contribution of the underneath substrate could be detected in this range (Fig. S1). To assess the specificity of this interaction, intact ZnO NRs were incubated with cells, and the peak at 360 nm is maintained, indicating that PBMCs do not interact with ZnO NRs. The inability of ZnO NRs to bind cells shows that our film preserves some specificity towards the selected antibody.

To further confirm that mAb was binding the rods, the surface of the film was analysed by SEM (Fig. 3b). When comparing the modified surface with that of the bare film (Fig. 1a), a completely different morphology can be depicted. The well-ordered and defined morphology assigned to the rods (Fig. 1a) can no longer be seen, and instead a dense layer associated to a protein coating can be observed on the NRs surface (Fig. 3b).

To characterize the interaction between ZnO NRs and mAb, a simple strategy using model proteins was used. ZnO NRs film were immersed in a solution of cytochrome C (Cyt C), a basic protein (isoelectric point, IEP ~ 10.7); myoglobin (Myo), a neutral reference protein (IEP ~ 6.9); or human serum albumin (HSA), an acidic protein (IEP ~ 5.9)^35^. After removing the ZnO NRs films from the solutions, the immobilization of the proteins on the surface of the rods was analysed by FTIR-ATR. The organic bonding (Fig. 3c) revealed intense vibrational bands at 794 cm^−1^, 1,014 cm^−1^, 1,086 cm^−1^, 1,257 cm^−1^, 1725 cm^−1^, corresponding to a rearranged ITO/PET substrate (Fig. S2), suggesting that the ZnO electrodeposition caused some chemical rearrangement on the underneath substrate. A faint band was observed at 840 cm^−1^, corresponding to the ZnO stretching mode. The band region between 1,550 and 1,660 cm^−1^ can be assigned to the stretching vibration modes of C–C, C=C and C–O. The ATR spectrum of ZnO NRs-Cyt C, like that of pristine ZnO NRs, suggests that no interaction took place with ZnO. By contrast, in the presence of either Myo or HSA a new peak appears at around 1,650 cm^-1^ (Fig. 3c). This peak, typical of the amide I region of proteins, indicates that Myo or HSA can bind ZnO NRs.

Since the global positive charge of Cyt C at neutral pH disfavoured the protein interaction with ZnO NRs, and, on the contrary, the global negative charge of HSA, and, to a lesser extent, that of Myo, favoured such interaction, one can hypothesise that an electrostatic interaction of the negatively charged region of mAb is made towards the positive face of Zn atoms of the top surface of the NRs, whereas the opposing face, bearing negative O atoms, is located towards the base were these NRs grow from, on the ITO/PET substrate (Fig. 3d). This result agrees with the well-described positive charge of ZnO, at neutral pH, where proteins with lower IEPs behave like negatively charged species, which, by leading to electrostatic interactions between ZnO and the proteins, result in their physical binding^36^.

This rod structure is particularly relevant for the optimization of the mAb binding sites. In fact, Wang et al. have concluded that ZnO NRs provided more protein interactions than other ZnO nanostructured materials^37^, reinforcing the development of ZnO NR-based immune-dependent platforms. For instance, Cao and colleagues have developed ZnO NRs-based sensors to immunodetect C-reactive protein, where a colloidal dispersion technique was used to cover PET^4, 38^. Similarly, Tamashevski et al.^15^ developed a ZnO NRs-based sensor towards the immunodetection of human leukemic cells, where gaseous-dispersive synthesis was used to cover a glass substrate. Both approaches resulted in randomly distributed ZnO NRs, which, due to their structural nature, cannot be optimized to favour the exposure of the desired polar faces.

In our work, by using electrodeposition, it was possible to assemble a well-organized ZnO NR film (Fig. 1a,b) that provides the optimal morphology towards the maximization of mAb immobilization. This strategy, boosting the electrostatic interactions between the rods and the mAb, obviates the need for an extra step of immobilization that often involves a covalent modifier^36^. This can be of relevance if, instead of biosensing, the aim is to recover the selected entities.

**Figure 4:**
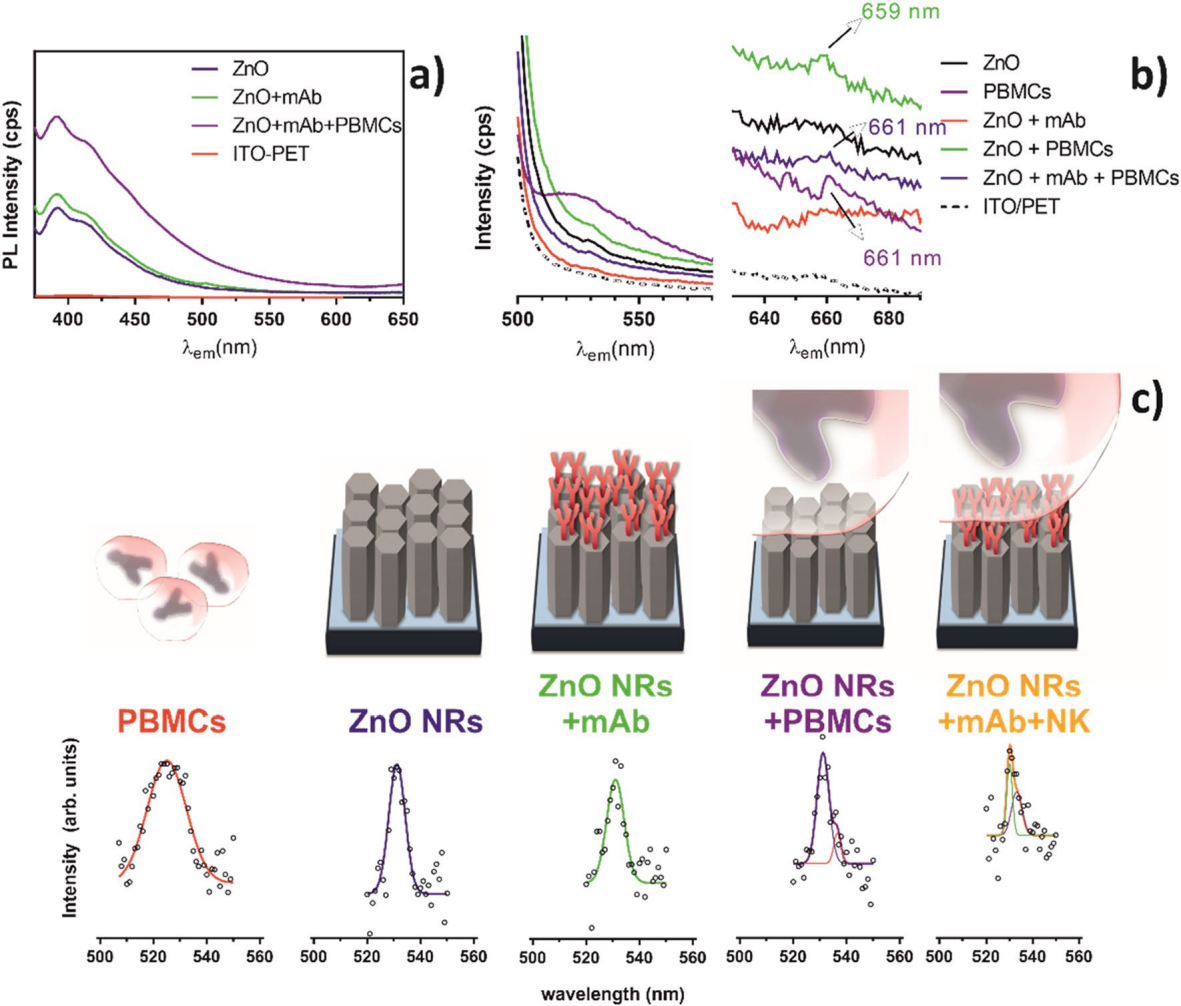
Characterization of the affinity of the ZnO NRs film to the antibody and PBMCs by photoluminescence (PL). Photoluminescence spectra with excitation at (**a**) 360 nm and (**b**) 483 nm are shown. (**c**) deconvolution of the PL spectra at 483 nm in the green emission range; the deconvolution in the red emission range is presented as Fig. S1.

Upon the successful design of ZnO NRs, the integrity of the film along the incubation process was assessed. To do so, photoluminescence (PL) spectra of ZnO NRs films were recorded at the excitation wavelength of 360 nm (Fig. 4a), which corresponds to the characteristic absorption band of ZnO. An intense peak at 393 nm in the UV–VIS region, along with another band at 415 nm, was observed. The PL spectra of ZnO nanostructures generally exhibits near-band-edge (NBE) emissions and broad deep-level emission (DLE) or visible luminescence due to exciton transitions and defect emission, respectively^39^. The emission peaks in the visible region at 415 nm can be tentatively attributed to the electron transitions from interstitial zinc (Zn_i_) related defect levels to valence band^40^. The origin of the DLE band is still a matter of discussion that derives from the fact that it depends on several factors, such as crystal perfection, doping, impurity availability, and surface morphology^41, 42^. Moreover, recent reports showed that different areas of ZnO structures may contribute differently to the PL spectra. Recently, data from room temperature cathodoluminescence of ZnO microrods indicated that the DLE is highly complex and occurs from side facets, whereas the UV emission originates mainly from the top planes of microrods^43^.

From the PL spectra at 360 nm (Fig. 4a) one can observe that no significant change in the intensities of PL peaks occurs upon mAb addition. Nevertheless, an increase in PL intensity was observed upon AO-loaded PBMC addition to the antibody immobilized ZnO system, reinforcing that these cells interact with our ZnO NRs-mAb system, allowing for their subsequent detection. The presence of the ZnO peaks along the various steps of antibody immobilization and cell detection (Fig. 4a) confirmed the stability of the electrodeposited film and, hence, of our nanoplatform.

To investigate the ability of our system to detect NK cells, a cell suspension was incubated with acridine orange (AO), and spectra were recorded at 483 nm, the excitation wavelength of AO (Fig. 4b). A broad intense band was observed when analysing AO-loaded PBMCs. At the excitation wavelength of AO, small narrower bands appeared on the spectra obtained with unmodified ZnO NRs films, with mAb-bound films, in the presence of AO-loaded PBMCs and also with mAb-bound films in the presence of AO-loaded cells (Fig. 4b).

Since no interference of the substrate exists at this excitation wavelength (Figure S2), and in order to identify the role of each player in our system, bands were deconvoluted into a sum of Gaussian functions to assess individual contributions (Fig. 4c). The presence of AO inside PBMCs was detected by two bands depicted at 525 and 661 nm. These two emission bands correspond to AO molecules binding to DNA and RNA, respectively^23, 24^. This observation contrasts with the single band detected for the ZnO film before or after mAb immobilization (apart from the initial dispersion of excitation light) at 531 nm (Fig. 4c). These results indicate that the two non-overlapping bands in the green emission range can be used to detect AO emission on ZnO NRs films. The AO spectrum resulting from AO-loaded cells is modified when in the presence of the ZnO film, either prior to or after mAb immobilization (Fig. 4c). In both situations, the band of ZnO NRs film was kept at around 531 nm, whereas two different sets of bands at 537 and 659 nm, prior to mAb immobilization, and at 533 and 661 nm, after mAb immobilization, appeared. This data indicates that distinct AO interactions are occurring in our system.

In the case of ZnO NRs interaction with PBMCs, the proven inability of the film to bind these cells (Fig. 3a) shows that an interaction of the rods with AO may exist. This can arise from some cytotoxic effect of ZnO on PBMCs, as suggested by the band appearing at 659 nm, a red emission that can result from the presence of AO in primary lysosomes and phagolysosomes, often associated to apoptotic events^23^. Despite the well-described resistance of resting T cells to ZnO NRs when compared with activated T cells, some toxicity has been reported^44^. This effect, proposed to occur through apoptosis, results in AO release to the medium. Once in the medium, AO can be adsorbed on the NRs, at an optimal pH of 7, as proposed for the ZnO mediated photocatalytic degradation of this dye^45, 46^, or, due to the green emission of this system, at 537 nm, by the direct binding of free DNA-AO to the rods^47^.

A green emission shift, that occurs when the antibody is present, suggests that the ZnO NRs-mAb interaction has a dual role in our nanoplatform. The presence of the antibody, by forming a protective protein layer (Fig. 3b), prevents cells from contacting the rods and acts as a selector towards NK cell binding. This role, successfully recorded by the band emission at 533 nm (Fig. 4c), confirms the presence of NK cells, and, together with the small contribution in the red region at 661 nm, suggests the presence of non-apoptotic cells (Fig. 4b).

**Figure 5:**
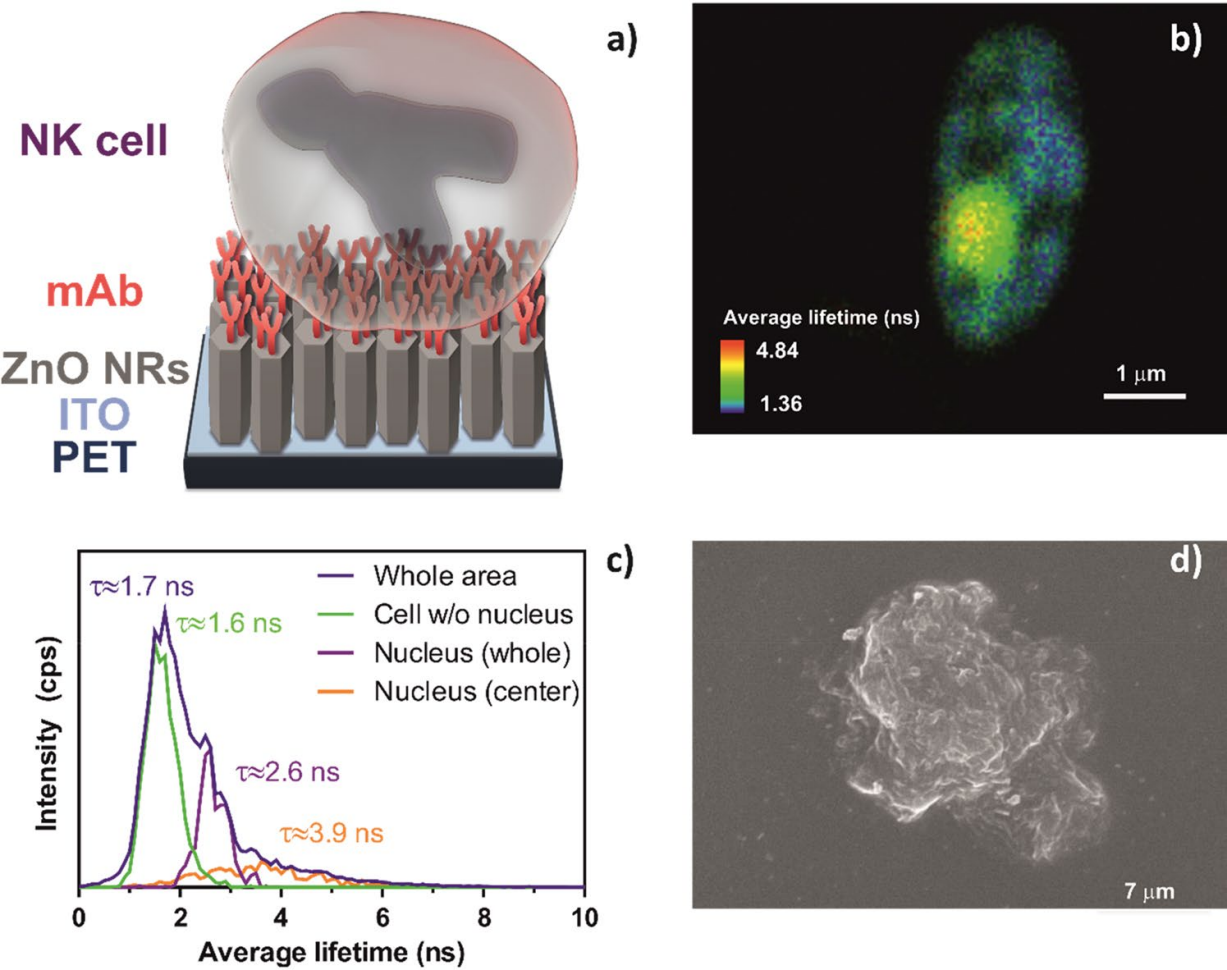
Characterization of PBMCs immobilized on ZnO NRs-mAb nanoplatform. (**a**) Scheme of our system; (**b**) fluorescence lifetime imaging microscopy (FLIM) image of an immobilized PBMC, and (**c**) corresponding lifetime histograms; (**d**) scanning electron microscopy (SEM) image of a ZnO NRs-mAb immobilized PBMC.

To further confirm the successful immobilization of NK cells in our ZnO NRs-mAb nanoplatform (Fig. 5a), a detailed analysis of the cells was performed. By using AO as the fluorescent reporter in stained cells, and laser excitation at 483 nm, FLIM was used to provide further details about the immobilized cells. As shown in Fig. 5b, a clear image of a PBMC was obtained upon immobilization. This oval-shaped object of 5.0 × 2.5 µm^2^ presented a lifetime histogram with short lifetimes together with a heterogeneous distribution of the dye throughout the cell (Fig. 5c). Such short lifetimes can be linked to the prevalence of AO monomers, indicating intercalation with nucleic acid molecules. SEM analysis (Fig. 5d) revealed that the immobilized PBMCs displayed their typical round-like shape^32^.

These findings show that cell morphology was not affected by their immobilization on our ZnO NRs-mAb nanoplatform. This affords new possibilities not only in terms of biosensing but also towards the recovery of these cells for possible transplantation therapies.

## Conclusions

The use of electrodeposition on a flexible ITO/PET substrate resulted in the deposition of well-ordered ZnO NRs, adequate for mAb immobilization and NK cell selection. The successful immobilization of the antibody, achieved by the electrostatic interaction with the ZnO nanorods, obviates the use of extra procedures to promote binding, which can convey some limitations when aiming to recover the selected cells. Overall, this approach allowed the production of high quality and affordable ZnO NRs films, and represents a step forward in the development of new and effective flexible nanoplatforms for immunoselection. The positive identification of NK cells, by coupling a specific NKp30 agonist, B7-H6, with acridine orange (AO), allowed us to assess the specificity of our nanoplatform. By using a mouse anti-human anti-NKp30 monoclonal antibody, the specificity towards NK cells was attained, with the mAb immobilization strategy used in our system preventing the loss of this mAb-based recognition. The challenge of immobilizing large entities as NK cells was successfully overcome by using the new ZnO NRs-mAb nanoplatform presented herein, maintaining an healthy cell morphology. This conveys the possibility of using this nanoplatform not only for biosensing, but also to recover these powerful host-immune cells.

## Methods

### Production of ZnO NRs film

**Figure 6:**
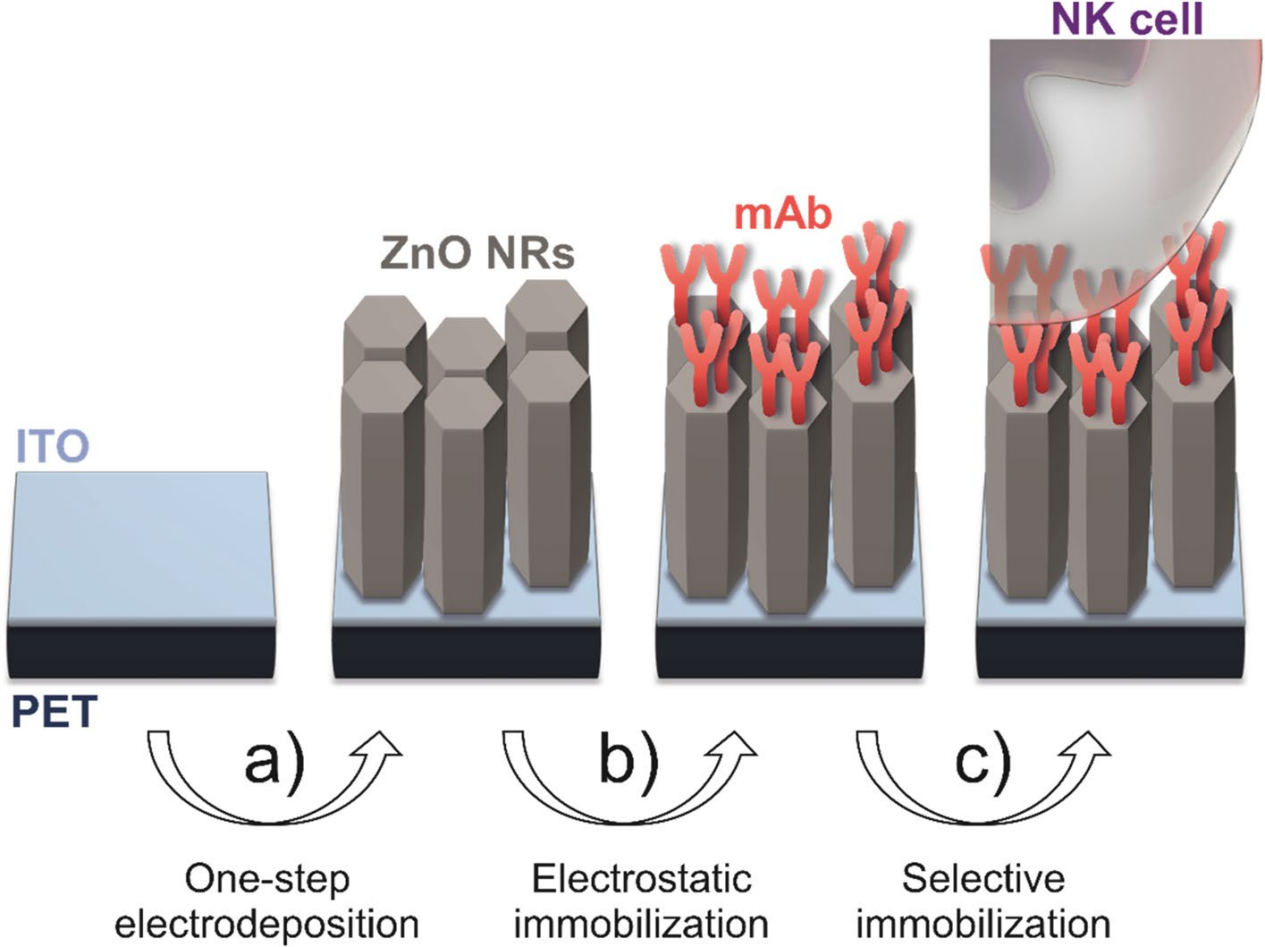
Representation of the experimental setup for ZnO NRs-mAb-PBMC assembly. (**a**) ZnO NRs were grown on ITO/PET by a one-step electrodeposition procedure; (**b**) the immobilization of mAb on the ZnO NRs was performed by a simple electrostatic immobilization; (**c**) the selective immobilization of PBMCs was made via mAb.

Vertically aligned ZnO nanorods were deposited on ITO-coated PET flexible substrate (ITO/PET) (from Solems, 80 nm thickness), using an electrodeposition technique (Fig. 6a). For that purpose, a three-electrode classic cell was used with a saturated calomel electrode (SCE) as the reference electrode, ITO/PET as the working electrode (WE), and a platinum plate as a counter electrode (CE). Before use, ITO/PET was cleaned with acetone and ethanol in an ultrasonic bath, for 5 min with each solvent, and rinsed with distilled water. The electrolyte solution used contained 5 mM ZnCl_2_ (Sigma Aldrich, purity > 95%) and 1 M KCl (Sigma Aldrich, purity 99.0–100.5%). The electrodeposition was carried out with an Autolab potentiostat (PGSTAT101) by applying a constant cathodic voltage of − 1.7 V vs SCE, for 30 min, at 70 ºC (Cryostat bath).

### Physicochemical characterization of NRs

Scanning electron microscopy (SEM) morphological analyses were performed on a JEOL-JSM7001F or a Hitachi S2400 microscope. Whenever required, the conductivity of the samples was improved with a thin gold/palladium coating (Polaron E-5100). For the transmission electron microscopy (TEM) studies, a Hitachi H-9000-NA microscope, operating at 200 kV, with supporting copper-carbon grids, was used, and the elemental chemical composition was assessed by the corresponding X-ray energy dispersive spectrometer (EDS). The crystallinity of particles was assessed by X-ray diffraction (XRD) using a D8 Advance Bruker AXS θ-2θ diffractometer with a copper radiation source (Cu Kα, λ = 1.5406 Å) and a secondary monochromator operating at 40 kV and 30 mA. UV–vis spectra were recorded on a Perkin Elmer Lambda 35 spectrophotometer.

### Protein binding

Solutions of myoglobin (Myo, from horse skeletal muscle, Sigma), human serum albumin (HSA, Sigma) or cytochrome C (Cyt C, from horse heart, Sigma), at 1 mg/mL, were prepared with MilliQ water and were used to immerse ZnO NRs films overnight at room temperature. After immersion, samples were washed 3 times with *ca.* 1 mL of MilliQ water and dried at room temperature. For the characterization of the protein organic groups interacting with the ZnO NRs, Fourier transform infrared attenuated total reflectance (FTIR-ATR) spectra were acquired using a Nicolet (Thermo Electron) spectrometer.

### Photoluminescence and fluorescence measurements

A Perkin Elmer Lambda 35 spectrophotometer was used for UV–Vis measurements. Photoluminescence (PL) spectra were recorded at room temperature on a SPEX Fluorolog (Horiba Jobin Yvon) spectrofluorometer, using a 450 W Xe lamp as the excitation source and a solid sample holder to collect light at 22.5°, minimizing stray and reflected light from the sample surface. Fluorescence lifetime imaging microscopy (FLIM) measurements were performed on a time-resolved confocal microscope (MicroTime 200, PicoQuant GmbH)^48^. FLIM measurements of ZnO nanorods were made by taking random images (80 × 80 µm^2^) throughout the coverslip; in each of these, another set of random (10 × 10 µm^2^) images was also recorded. Briefly, excitation at 405 nm and 483 nm was carried out using a pulsed diode laser head at a repetition rate of 20 MHz. Permanent online analysis of the back reflects and backscatter excitation light was achieved with a CCD camera, monitoring the image seen by the objective. Band-pass filters 480AF30 transmission and long-pass filters 510LP transmission were used. Fluorescence lifetimes were detected with a single-photon avalanche diode (SPAD) (PerkinElmer), and the signal was processed by a Time Harp 200 TC-SPC PC board (PicoQuant) working in time-tagged time-resolved (TTTR) operation mode. For point-by-point measurements, fluorescence decays of > 30 pixel points were collected^31^. The peaks position on the PL spectra in the green and red emission ranges were deconvoluted into one or more components through the Gaussian curve-fitting method used in Origin 8.0 (OriginLab Corporation).

### System assembly

All the ZnO NR systems used in this study have the same total area (2 × 1 cm^2^). To assemble the antibodies on the ZnO NRs surface (Fig. 6b), 0.5 ng of monoclonal anti-human anti-NKp30 antibody (Abcam, UK) (50 µL of a 10 µg/mL solution in PBS) were added onto the surface of ZnO NRs and dried overnight at 4 ºC in a desiccator.

### Peripheral blood mononuclear cells (PBMC) isolation and acridine orange (AO) loading

This study was approved by the Comissão de Ética para a Investigação com Seres Humanos (CEISH) of Faculdade de Farmácia, Universidade de Lisboa, as part of the "Precision Oncology by Innovative Therapies and Technologies” project (FCT funding SAICTPAC/0019/2015). All the manipulation of human samples was performed in accordance with the relevant guidelines. All participants provided written informed consent and personal data protection was safeguarded in all instances. Human venous blood (20 mL) was collected by venepuncture and mixed with 200 µL of 0.5 M sodium EDTA by gentle inversion. All samples were processed within 15 min after collection. Blood samples were layered on 20 mL of Ficoll Histopaque-1077 in 50 mL conical tubes and centrifuged at 400×*g* for 30 min at room temperature in a swing-out bucket rotor. The top serum layer was discarded and the buffy coat (PBMCs) was transferred into clean 50 mL conical tubes and washed three times with 20 mL of PBS containing 2 mM EDTA. Isolated PBMCs were resuspended in sterile human AB serum (Sigma-Aldrich) to a density of 4 × 10^6^ cells/mL with 10%(V/V) DMSO and cryopreserved (− 140 ºC, liquid-nitrogen gas phase). The average yield was 2 × 10^6^ cells/mL of blood. Frozen PBMCs were thawed in a 37 ºC water bath. The cryoprotectant (DMSO) was immediately removed by washing with 2 × 10 mL of sterile PBS containing 5% (m/v) BSA. Cell suspensions were adjusted to 1 × 10^6^ cells/mL in PBS 5% (m/v) BSA before use.

For acridine orange (AO) staining, cells were loaded with AO prior to being used by incubating 1 mL of a cell suspension (*ca*. 8** × **10^5^ cells/mL) with 1 µL of a 1 mg/mL AO solution in DMSO for 30 min at 37 ºC without shaking. Cells were harvested and washed 3 times with 1 mL of PBS supplemented with 5% (m/v) BSA and resuspended in 1 mL of the same buffer. This cell suspension was used for both the NK cells activation procedure and for the ZnO-mAb nanoplatform loading.

The activation of NK cells was performed by adding 3.5 µg/mL of B7-H6 (R&D Biosystems, USA) from a PBS stock solution to a 250 µL PBMCs suspension. B7-H6 is a specific NKp30 agonist that will trigger the activation of NKp30-expressing natural killer (NK) cells. Activated NK cells will undergo degranulation and, when pre-loaded with AO, the degranulation can be followed by tracking the fluorescent dye as well as these cells morphology.

For the nanoplatform loading 250 µL of PBMCs suspension (*ca.* 2 × 10^5^ cells) were used in each addition to the ZnO NR-coated ITO/PET support (Fig. 6c).

## Supplementary information

Supplementary Information 1.
