# Author Correction: Flexible ZnO-mAb nanoplatforms for selective peripheral blood mononuclear cell immobilization

**DOI:** 10.1038/s41598-021-03382-w

**Published:** 2021-12-07

**Authors:** K. Sowri Babu, Pedro F. Pinheiro, Cátia F. Marques, Gonçalo C. Justino, Suzana M. Andrade, Marta M. Alves

**Affiliations:** 1 Division of Physics, Dept. Of Science and Humanities, Vignan’s Foundation for Science, Technology & Research (Deemed To Be University), Vadlamudi, Guntur, AP 522213 India; 2 Centro de Química Estrutural, Instituto Superior Técnico, Universidade de Lisboa, Av. Rovisco Pais, 1049-001 Lisboa, Portugal

Correction to: *Scientific Reports* 10.1038/s41598-020-72133-0, published online 14 September 2020

The original version of this Article contained an error in the Acknowledgments section.

“This work was funded by Fundação para a Ciência e a Tecnologia through projects UID/QUI/00100/2019 and UIDB/00100/2020 to Centro de Química Estrutural, through research grants SAICTPAC/0019/2015 and PTDC/QUI-QAN/32242/2017, and through PhD grants SFRH/BD/110945/2015 (P.F.P.) and SFRH/BD/143128/2019 (C.F.M.). K.S.B. thanks EMBO for providing the short-term fellowship (EMBO ref. 8107). M.M.A. and G.C.J. are IST-ID employees under contracts IST-ID/154/2018 and IST-ID/090/2018, respectively, in agreement with D.L. 57/2017. The authors would also like to thank Ana Charas for providing the ITO/PET substrate.”

now reads:

“This work was funded by Fundação para a Ciência e a Tecnologia through projects UID/QUI/00100/2019 and UIDB/00100/2020 to Centro de Química Estrutural, through research grants LISBOA-01-0145-FEDER-016405 – SAICTPAC/0019/2015 and PTDC/QUI-QAN/32242/2017, and through PhD grants SFRH/BD/110945/2015 (P.F.P.) and SFRH/BD/143128/2019 (C.F.M.). K.S.B. thanks EMBO for providing the short-term fellowship (EMBO ref. 8107). M.M.A. and G.C.J. are IST-ID employees under contracts IST-ID/154/2018 and IST-ID/090/2018, respectively, in agreement with D.L. 57/2017. The authors would also like to thank Ana Charas for providing the ITO/PET substrate.”

The original Article has been corrected.

