# Supplementary material for: Flexible ZnO-mAb nanoplatforms for selective peripheral blood mononuclear cell immobilization
Source: Sci Rep. 2020 Sep 14;10:15018. doi: 10.1038/s41598-020-72133-0 (PMC7490409; doi:10.1038/s41598-020-72133-0)
Supplement: Supplementary file 1 — Supplementary Information 1. [file 41598_2020_72133_MOESM1_ESM.docx]

**Flexible ZnO-mAb nanoplatforms for selective peripheral blood mononuclear cell immobilization**

Sowri B. Kotika^a^, Pedro F. Pinheiro ^b^, Cátia F. Marques^b^, Gonçalo C. Justino^b*^, Suzana M. Andrade^b*^, Marta M. Alves^b,*^

**Supplementary information**


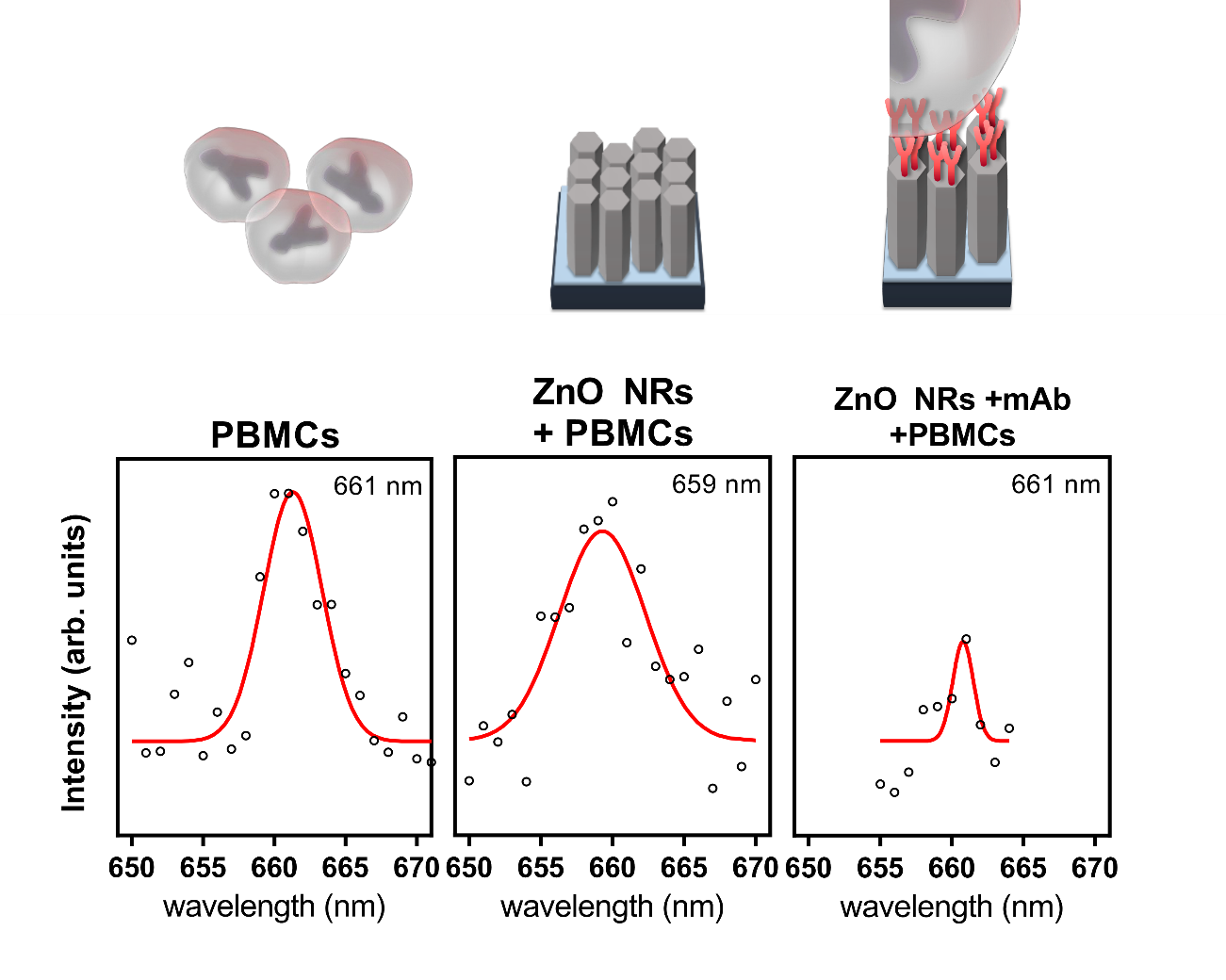


**Figure S1.** Characterization of the affinity of the ZnO NRs film to the antibody and PBMCs by photoluminescence (PL); deconvolution of the PL spectra at 483 nm (Fig. 4 b) in the red emission range.

**
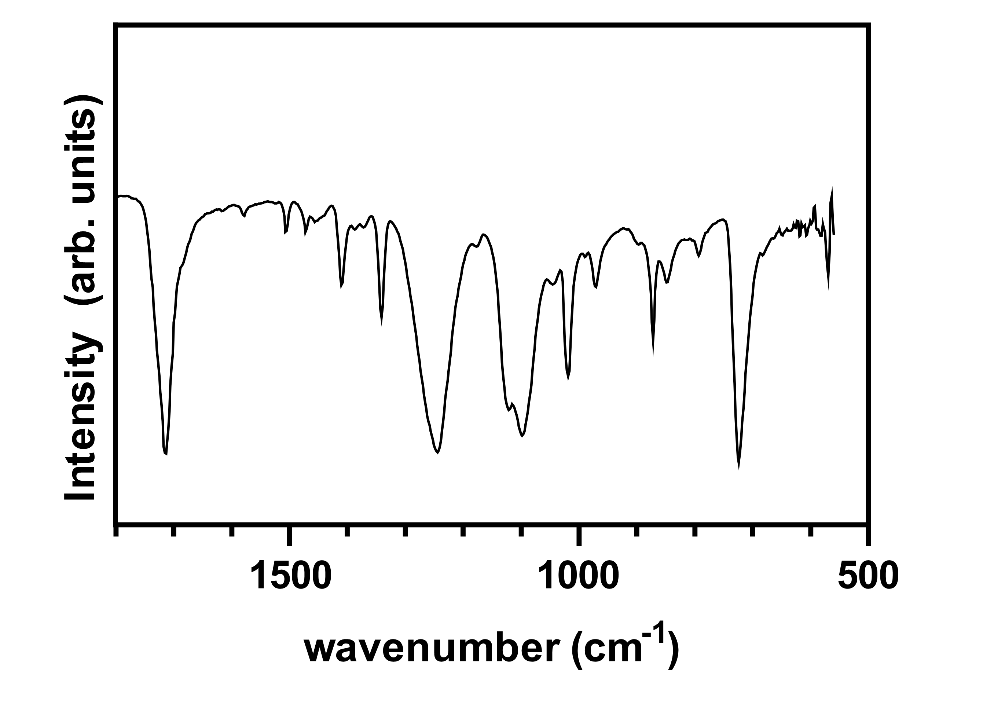
**

**Figure S2. Characterization of the PET-ITO substrate prior to electrodeposition - Fourier transform infrared attenuated total reflection (FTIR-ATR).**
